# Supplementary material for: Enhancing spontaneous recovery after stroke: a randomized controlled trial
Source: Brain Commun. 2026 Mar 28;8(2):fcag057. doi: 10.1093/braincomms/fcag057 (PMC13098183; doi:10.1093/braincomms/fcag057)
Supplement: fcag057_Supplementary_Data [file fcag057_Supplementary_Data.zip › FigureS1.pdf]

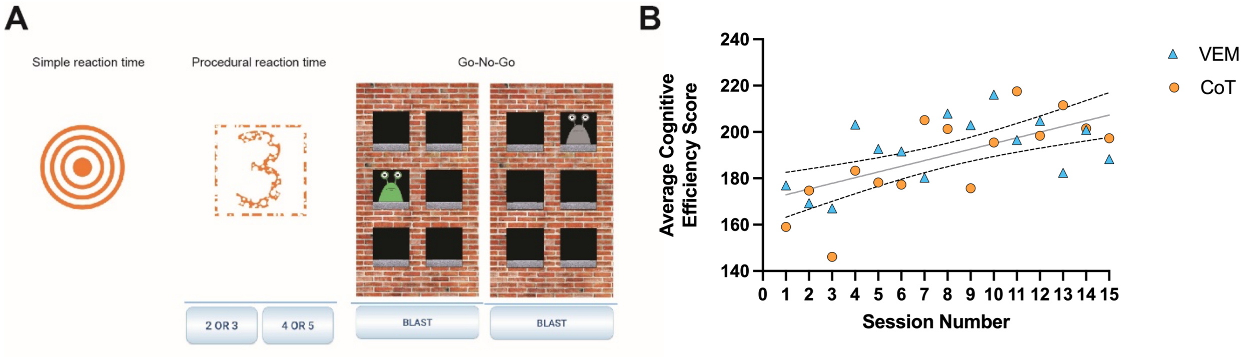

Figure S1 Cognitive efficiency was determined from performance of three reaction time (RT) tasks (A) completed on a smartphone running the DANA Brain Vital app during rest breaks within each of the 15 therapy sessions, which comprised the interventions. (B) Linear regression indicated participants across both VEM (N=24) and CoT (N=30) from per protocol analysis tended toward greater cognitive efficiency (faster RT) over therapy sessions. Each data point reflects the mean score of the group. Solid Line y = 2.46x + 170.5, Dashed lines = 95%CI, $F_{2,26} = 1.61$, $P = 0.28$, indicates one curve for both groups.
