# Supplementary figures and images for: Enhancing spontaneous recovery after stroke: a randomized controlled trial
Source: Brain Commun. 2026 Mar 28;8(2):fcag057. doi: 10.1093/braincomms/fcag057 (PMC13098183; doi:10.1093/braincomms/fcag057)

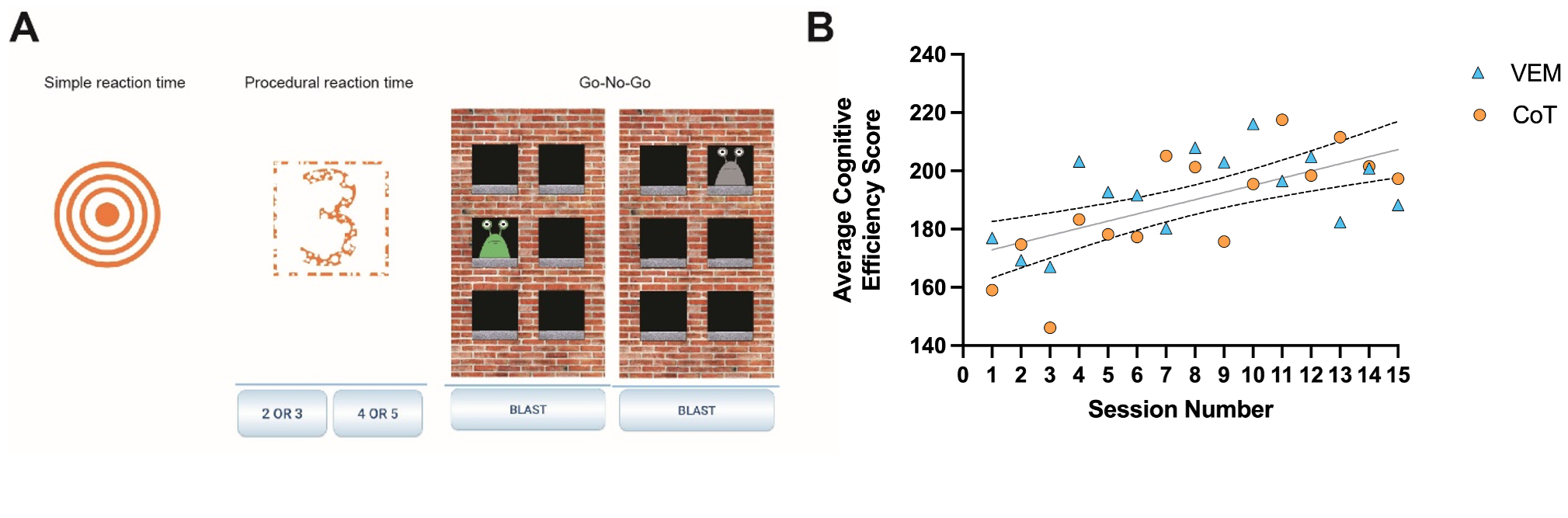

Supplement: fcag057_Supplementary_Data [file fcag057_Supplementary_Data.zip › FigureS1.tif]
